# Microbiological Food Safety of Seaweeds

**DOI:** 10.3390/foods10112719

**Published:** 2021-11-06

**Authors:** Trond Løvdal, Bjørn Tore Lunestad, Mette Myrmel, Jan Thomas Rosnes, Dagbjørn Skipnes

**Affiliations:** 1 Nofima–Norwegian Institute of Food, Fisheries and Aquaculture Research, Department of Process Technology, Richard Johnsens Gate 4, P.O. Box 8034, NO-4021 Stavanger, Norway; thomas.rosnes@nofima.no (J.T.R.); dagbjorn.skipnes@nofima.no (D.S.); 2 Institute of Marine Research, Section for Contaminants and Biohazards, Nordnesgaten 50, P.O. Box 1870, NO-5005 Bergen, Norway; bjorn-tore.lunestad@hi.no; 3 Virology Unit, Faculty of Veterinary Medicine, Norwegian University of Life Sciences, Elizabeth Stephansens vei 15, P.O. Box 5003, NO-1433 Ås, Norway; mette.myrmel@nmbu.no

**Keywords:** seaweed, macroalgae, food safety, microbiology, bacteria, viruses, seafood, foodborne disease, spoilage, food quality

## Abstract

The use of seaweeds in the human diet has a long history in Asia and has now been increasing also in the western world. Concurrent with this trend, there is a corresponding increase in cultivation and harvesting for commercial production. Edible seaweed is a heterogenous product category including species within the green, red, and brown macroalgae. Moreover, the species are utilized on their own or in combinatorial food products, eaten fresh or processed by a variety of technologies. The present review summarizes available literature with respect to microbiological food safety and quality of seaweed food products, including processing and other factors controlling these parameters, and emerging trends to improve on the safety, utilization, quality, and storability of seaweeds. The over- or misuse of antimicrobials and the concurrent development of antimicrobial resistance (AMR) in bacteria is a current worldwide health concern. The role of seaweeds in the development of AMR and the spread of antimicrobial resistance genes is an underexplored field of research and is discussed in that context. Legislation and guidelines relevant to edible seaweed are also discussed.

## 1. Introduction

The global seaweed industry is worth more than USD 6 billion per year, corresponding to approx. 12 million tons/year in volume, of which about 85% comprises food products for human consumption [1]. Owing to the fact that there will be an increasing need for protein food sources to accommodate the anticipated growth in the world’s population, the seaweed industry (both aquaculture and wild-harvested) is expected to grow since seaweed is a sustainable food source. This assumed increase, together with consumers’ demands for tasty, nutritious, safe, and convenient seaweed food products, and changes in market trends, leads to a growing need to ensure microbially safe seaweed food products.

Several studies have focused on the bacterial diversity in brown (Phaeophyceae), green (Chlorophyta), and red (Rhodophyta) macroalgae (henceforward: seaweed). Bacteria inhabiting seaweed include the Proteobacteria, Actinobacteria, Bacteroidetes (CFB group), Cyanobacteria, Firmicutes, Planctomycetes, Verrumicrobia, Chloroflexi, Deinococcus-Thermus, Fusobacteria, and Tenericutes, with the Gammaproteobacteria as the most common bacterial clade [2,3,4,5]. However, there are only a few studies that specifically clarify the prevalence of human pathogens in edible seaweeds [6,7,8]. 

The total number of bacteria varies according to season and is typically lowest during spring and among younger plants [9,10,11], but this may also be species- and location-dependent. Although the density and composition of bacteria on seaweed are strongly correlated to that of the surrounding water, it is frequently reported that the microbiota associated with seaweed is different from what is found in the seawater in which they grow [2,12]. A relatively specific bacterial flora can be found to associate with different phyla of marine seaweed growing in the same habitat [8,13].

The viable counts reach up to log 7 bacterial cells per gram of seaweed biomass when using agar spread plate methods and are shown to be higher when applying direct (microscopy-based) techniques (Table 1). 

After the first impression formed by aroma, color, and general appearance, the number of microorganisms on the fresh edible seaweeds may serve as a secondary indicator for the food quality and safety of the edible seaweed, but not more so than for fruits and vegetables, which can have comparable bacterial loads on their surfaces. A high bacterial count of seaweed is indicative of the age and health of the plant, but primarily of the microbial load and composition of the surrounding water masses. High initial bacterial loads normally affect the shelf life and sensorial quality of the product negatively, but do not necessarily imply that the food is unsafe to consume. On the other hand, a low bacterial number does not necessarily imply that it is safe. For some pathogens, especially for the toxin-producing bacteria, consumption of relatively small amounts is sufficient to cause severe health problems in humans, and even death. 

There is a general assumption that human pathogens occur on seaweed in the same density and composition as in the surrounding water masses. Hence, the localization of the seaweed is an important factor concerning microbiological food safety [6,20,25,26]. However, seaweed food products may also get contaminated or re-contaminated during handling and processing [27,28,29]. Locations in coast-near areas with poor water quality may be predisposed to human pathogens. Researchers concluded that consumption of seaweeds collected in Danish waters is safe, as long as harbors and areas influenced by agricultural and industrial run-off are avoided [26]. A Norwegian study concluded that—although seaweed is densely covered by bacteria, including potential pathogens that may be challenging during processing or improper storage—the risk of macroalgae as the origin of foodborne diseases cannot be expected higher than for other non-filtering marine organisms, including fish [7].

The increasing use of antimicrobials in, e.g., aquaculture has led to concerns about the development of antimicrobial resistance (AMR) in bacteria and the spread of antibiotic resistance genes (ARG) and that it may compromise successful treatment of bacterial infections [30]. The presence of resistant bacteria in the human food supply chain is documented [31], but the role of seaweed is not yet clear. This represents a data gap that warrants more research.

This review is restricted to studies of microbiological food safety of marine seaweed belonging to the brown, green, and red algae. Antimicrobial properties of seaweeds, their derived extracts, or microbial symbionts, are not covered in the present review, nor nutrition or sensory aspects of edible seaweed. The review focuses on human pathogens that may challenge food safety, and not pathogens that may exclusively be detrimental to the plant itself. 

## 2. Pathogenic Microorganisms in Seaweed

Bacteria, viruses, yeast, and molds may constitute potential microbiological health hazards in edible seaweed. Regarding bacteria, separation is made between (i) pathogenic bacteria that may be present in such small amounts that it does not lead to a directly observable effect (flavor, color, aroma) of the product, but as by ingestion of minute quantities may still cause food poisoning and even death, and (ii) spoilage bacteria, which is not necessarily harmful to the consumer, but which degrade the product. The main factors for bacterial contamination of seafood are contamination of the raw material from the environment and from the processing, and bacterial growth conditions. The following Section deals with pathogenic microorganisms associated with edible seaweeds. The specific processing factors that are relevant for seaweed in the frame of food safety and quality, are discussed in more detail in Section 3. 

### 2.1. Bacillus sp.

More than 140 species are at present included in the genus *Bacillus* [32], and they are commonly described as Gram-positive, rod-shaped, straight, or slightly curved cells, that appear singly, in pairs, chains, or as long filaments. They are further referred to as possessing the ability to form resistant endospores, one per cell, although sporulation remains to be documented in some of the recently described species. *Bacillus* spp. are commonly aerobic, but some species are facultatively anaerobic, and at least two strictly anaerobes have been described. Although the majority of the species belonging to the genus *Bacillus* have little or no pathogenic potential, some species are known to be associated with food-borne diseases in humans, by means of the production of heat-stable toxins. *B. cereus* may cause food poisoning and opportunistic infections, while some other species, including *B. subtilis*, *B. pumilus*, and *B. licheniformis*, have also been associated with food poisoning and human/animal infections [32,33,34,35,36]. 

*Bacillus* spp., among others, are efficient producers of compounds with antibacterial, antifouling, and quorum sensing inhibiting features, which make them highly successful colonizers of seaweed surfaces, and may live in an endosymbiotic relationship with seaweed [2]. Growth promoting and nutritional effects beneficial to the seaweed have been attributed to endophytic *Bacillus* spp., including *B. cereus, B. pumilus,* and *B. licheniformis*, and these species are associated with seaweed of the brown, green and red algae [37,38,39].

Concerns were raised about *B. cereus* in various dehydrated, ready-to-eat (RTE) seaweed products sold in Italy [24], *B. subtilis* on edible brown seaweed harvested off the coast of Ireland [40], *Bacillus* spp. in seaweed cultivated in Scotland [8], and *B. licheniformis* and *B. pumilus* on edible brown seaweed cultivated in Norway [21]. Although the concentrations of *Bacillus* spp. observed on fresh seaweeds may be low compared to what is considered as the infectious dose, measures need to be taken to control the growth of these species in the food during handling and storage. This was demonstrated by a probability distribution model for levels of *B. cereus* in RTE kimbab (rolled cooked rice and other foodstuffs in dried green seaweed) which estimated that contamination levels at the time of consumption ranged from −3.63 log cfu/g to 7.31 log cfu/g when the model parameters storage time (2.31 ± 4.63 h) and temperature (22.5 ± 3.17 °C) [41], and conservative initial *B. cereus* concentrations (−4.85–0.69 log cfu/g [undetectable]) [42] were based on relevant data surveyed from stores selling RTE kimbab in Korea [43]. Kimbab is a RTE type of take-away food that is typically prepared by hand and stored at room temperature, which is probably contributing strongly to contamination and growth. 

It is the *Bacillus* toxins that are the actual harmful agent, and not the bacteria themselves, so it is not straightforward to derive a generalized infective dose based on the contamination level. However, for *B. cereus*, *B. pumilus*, and *B. licheniformis*, concentrations needed to produce enough toxin to induce food poisoning is considered to be ≥log 5 cfu/g [33,34,44,45]. In relation to combinatorial food products with seaweed, as e.g., kimbab, contaminating bacteria (e.g., *Bacillus* spp. and *Staphylococcus aureus*), may well originate from e.g., rice or soybean paste, and not the seaweed [29].

**Figure 1:**
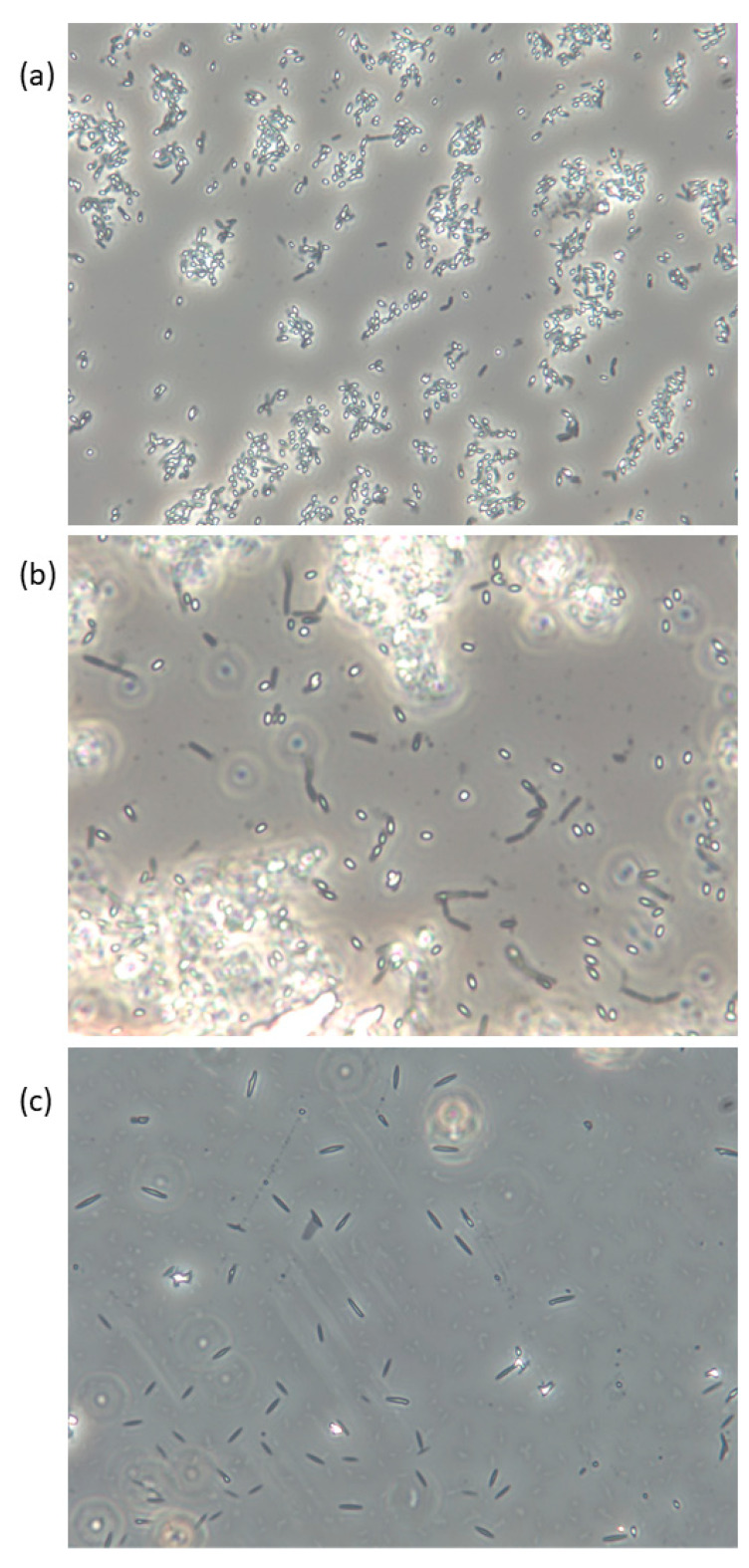
Live phase-contrast microscopy images of (**a**) *B. licheniformis*, (**b**) *B. pumilus*, and (**c**) *B. subtilis* isolated from *Saccharina latissima* and cultivated on Marine Agar. Spores appear white/bright and vegetative cells are dark. Magnification: 400×.

Spores of *Bacillus* spp., as exemplified in Figure 1, are very resistant to most external factors and can tolerate temperatures over 100 °C combined with pH < 3 for several minutes [46], but will not be able to reproduce under such conditions. Spores present in the product may on the other hand be able to germinate when the conditions allow and reproduce and eventually produce toxins that may lead to food poisoning and in the worst-case death. Table 2 summarizes limits for growth in relation to temperature, pH, water activity (a_W_), and water phase NaCl for some human pathogen spore formers in their vegetative form, in addition to some other potentially harmful bacteria associated with seaweed. The growth rate will decrease with lower temperatures and pH until their minimum limit is reached. A seaweed product may be considered safe to eat as long as pH is below 4.3 when stored at ≤4 °C (cf. *B. cereus*). If the product is to be stored at an elevated temperature, pH needs to be lowered to ≤3.7 (cf. *Salmonella*). *B. licheniformis*, *B. pumilus,* and *B. amyloliquefaciens/subtilis* are not able to grow or produce toxins at refrigerated temperatures (Table 2). 

### 2.2. Pathogenic Vibrios

Bacteria in the genus *Vibrio* are Gram-negative, curved rod-formed, and facultative anaerobes [60]. Members of the genus have the sea, brackish and fresh water as their natural habitat and are among the most common bacteria found in surface waters worldwide [61]. Considering the widespread prevalence of vibrios in aquatic environments, it is not surprising that seaweeds are frequently colonized by members of this genus [62]. There are currently over 140 *Vibrio* species, of which 12 are reported to be associated with infections among humans [63,64,65]. The most important human pathogenic species are *V. cholerae*, *V. parahaemolyticus,* and *V. vulnificus* [65,66], but also several other *Vibrio* species as *V. alginolyticus*, *V. metschnikovii*, *V. fluvialis,* and *V. mimicus* may cause infection but with less severe symptoms in humans [65]. The prevalence of human pathogenic vibrios and especially those possessing genes for increased pathogenicity are highly correlated with high water temperatures [67], and global warming is expected to favor their distribution [61]. As the vibrios are indigenous to the aquatic environment, there is no documented correlation between the occurrence of *Vibrio* and commonly applied indicator bacteria of fecal contamination. Thus, indicator organisms as coliforms do not give information on the presence of potentially pathogenic *Vibrio* spp.

Water and various foods have been implicated as vehicles for the highly pathogenic *V. cholerae* O1 and O139 as demonstrated by epidemiologic studies [68]. A very rare case was reported in which a woman acquired infection after eating raw, fresh seaweed transported from the Philippines as hand luggage to her home in California and eaten a month later [69]. However, *V. cholera* cannot be considered a likely pathogen associated with seaweeds.

Food poisoning caused by *V. parahemolyticus* and *V. vulnificus* associated with edible seaweeds also appears to be rare, but several documented examples from other kinds of seafood are known, e.g., prawns and oysters [70,71], the latter usually in immunocompromised individuals. Findings of *V. parahemolyticus* [72] and *V. vulnificus* [73] in seaweeds collected along the coast of Japan, prompted the authors to encourage proper hygiene practice during postharvest handling of seaweeds, especially in summer when the concentrations peaked. *Vibrio* spp. counts as high as log 8.2 cfu/g have been reported on raw cultivated *Gracilaria changii* harvested in Malaysia, indicating the potential presence of human pathogens possibly compromising food safety if consumed raw [20]. 

The vibrios are considered particularly sensitive to food processing, especially thermal treatment. However, in samples of sundried *Ulva lactuca* cultivated in Turkey, *Vibrio* spp. were reported in a number of <10 cfu/g [17]. Using sensitive qPCR assays combined with microbial pre-enrichment, Barberi et al., 2020 [74] detected pathogenic *V. parahemolyticus* in 78% of cultivated seaweed samples from North-East USA. Kudaka et al., 2008 [19] identified *V. parahemolyticus* in 18.8% of samples of *Caulerpa lentillifera* (Sea grape) cultivated in tanks. Although the thermostable hemolysin gene was not detected in any of the isolates, these findings led the authors to highlight the importance of a suitable sterilization process for *C. lentillifera* to ensure food safety [19]. *V. alginolyticus* was isolated from cultivated *A. esculenta* in Scotland, but not *V. vulnificus*, *V. parahemolyticus,* or *V. cholera* [8]. Conventional culturing methods failed to identify *Vibrio* spp. in seaweeds collected in Ireland [18] or Norway [21]. 

Ziino et al., 2010 [25] reported a high prevalence (75%) and relatively high densities (log 1.30–4.60 cfu/g) of *Vibrio* spp. in the traditional seaweed dish “mauro” (i.e., *Chondrus crispus* and *Chondracanthus teedii*) sold in Catania, Sicily, Italy, and eaten raw. The most frequently isolated species were *V. alginolyticus*, followed by *V. parahemolyticus*, *V. coralliitycus*, and *V. mimicus*, all of which included strains with genomes encoding one or more of the virulence genes ToxR, ToxRS, tlh, or trh. However, of these species, it is only *V. parahemolyticus* that is considered a food-borne human pathogen. As pointed out by the authors [25], the reason for the high amounts of potential pathogens, in this case, may be that the seaweed was collected in the height of summer in an area used for recreational activities causing anthropogenic contamination, again highlighting the importance of collecting seaweeds in unpolluted waters of a high quality. Furthermore, it cannot be ruled out that cross-contamination occurred during handling. 

Potentially pathogenic *Vibrio* species have occasionally been detected in the environment and seafood organisms from temperate waters [75], but seaweed has so far not been identified as a challenge regarding vibrios [7]. 

### 2.3. Aeromonas sp. 

The genus *Aeromonas* belongs to the family Aeromonadaceae, and is a group of Gram-negative, rod-shaped, oxidase- and catalase-positive and facultatively anaerobic bacteria [76,77]. Members of this genus are ubiquitous aquatic bacteria and thus common in environments such as fresh-, brackish and marine water, and also found as inhabitants of aquatic animals [77]. *Aeromonas* spp. are potential foodborne pathogens and known to cause gastrointestinal as well as extra-intestinal infections in humans [78]. Most studies have dealt with *A. hydrofila*, which have been implicated in many seafood-borne outbreaks [79]. The occurrence of *Aeromonas* spp. has been frequently reported in water and food, including RTE seafood [80,81,82]. Currently not much is known on the role of seaweeds as responsible food for infections. However, based on their indigenous aquatic prevalence, *Aeromonas* spp. could be expected to colonize seaweeds and possibly follow the raw materials to processing. Furthermore, the ability of some *Aeromonas* sp. to survive and even grow at chilled temperatures gives reason for concern for seaweed and other seafood products. *A. hydrophila* was isolated from e.g., *Ulva reticulata* harvested in Malaysia [83], and *Aeromonas* spp., in concentrations up to log 5.9 cfu/g, from mauro prepared from *Chondrus crispus* and *Chondracanthus teedii* sold by fishmongers or from street stalls in Sicily, Italy [25]. 

### 2.4. Escherichia coli, Salmonella spp., Listeria monocytogenes, Staphylococcus aureus, and Other Microorganisms Associated with Health Hazard in Seaweed 

Bacterial pathogens on seaweeds for human consumption may origin from two main sources; the environment in which they are grown and from equipment and humans who handle the algae after harvest. Pathogens from environmental and anthropogenic sources may persist in coastal waters and can potentially cause contamination. Research on bacterial pathogen contamination of seaweeds is limited, and literature is scarce for some areas e.g., US coastal waters [74] while more literature is found from other parts of the world. Sugar kelp *Saccharina latissima* and adjacent water were sampled from three sites of seaweed aquaculture located in adjacent bays of Maine, USA, during the winter growing season [74]. Membrane filtration onto selective media detected *E. coli* and *Vibrio* species in seaweed and water samples at all sites, however with very low plate counts. The foodborne pathogens *Salmonella enterica* ser. Typhimurium and enterohemorrhagic *E. coli* O157:H7 were detected on enriched seaweed samples from 83%, 78%, and 56% of sampling events, respectively, using molecular methods [74]. 

The Ministry of Environment and Food of Denmark proposed a guideline of 100 cfu/100 g of seaweeds for *E. coli*, as an indicator organism for fecal pollution, and a limit of none detected in 25 g for *Salmonella* [26]. The hygienic quality of edible seaweeds in Danish waters was assessed by analyzing 65 samples of brown (*Fucus vesiculosus, Fucus serratus, Fucus spiralis*) and green (*Ulva lactuca*, and *Cladophora* spp.) seaweeds distributed along the Danish coastline. The *E. coli* counts were above the proposed limit in eight samples of the brown seaweed *F. vesiculosus*, including two samples with >1000 and >3000 cfu/g, respectively, collected in proximity to agricultural run-off or harbor basins. *E. coli* in the remaining six samples served as a reminder of fecal pollution and possible association with norovirus [26]. *Salmonella* sp. was not detected in any of the 65 samples, prompting the conclusion that, as long as pollution sources and industrial run-off and harbors are avoided, it is safe to collect seaweeds for human consumption in Denmark, but it could not be concluded from the results where, geographically, it is safe [26]. 

A few European studies failed to detect gastrointestinal pathogens on wild-collected seaweeds, including *Laminaria* [7,18,40,84]. In a study on *Saccharina latissima* and *Alaria esculenta* farmed in Norway, no enterococci, coliforms, pathogenic *Vibrio*, or *Listeria monocytogenes* were found through plating methods [21]. *Salmonella, E. coli,* and *S. aureus* were absent in samples of cultivated *S. latissima* and *A. esculenta* collected in Scotland, but one sample of *A. esculenta* was positive for *L. monocytogenes*, probably as a result of cross-contamination during handling [8]. 

When analyzing RTE products that include seaweeds, the sources of contamination are more unknown and may have a cause in the failures of hygiene procedures. Cho et al., 2008 [85] examined 30 kimbab samples using a multiplex PCR method and found 83.3% of samples contaminated. The contamination rates were for *S. aureus* (56.7%), *B. cereus* (43.3%), *Salmonella* spp. (36.7%), *Shigella* spp. (13.3%) and *L. monocytogenes* (6.7%). An examination of 258 kimbab and lunch boxes showed 13.2% contamination and *S. aureus*, *B. cereus,* and *Yersinia enterocolitica* were identified [29]. *S. aureus* is frequently found in kimbab in concentrations up to 3.5 log cfu/g [29,42,85]. In risk assessments of *S. aureus* for kimbab, a maximum storage time of 5–7 h at ambient temperatures is recommended, dependant on initial numbers of *S. aureus*, time-temperature relationship, and other growth factors [86]. 

Besides the bacteria mentioned above, the following microorganisms are associated with health hazards in seaweed: 

(i) *Campylobacter jejuni* and *Yersinia enterocolitica* can be isolated from water and seafood but are not reported as a serious health hazard in edible seaweed. The former is very sensitive to NaCl and other environmental factors, and it is mostly non-pathogenic strains of the latter that are isolated from the environment. *Y. enterocolitica* was detected in less than 1% of kimbab samples, and *C. jejuni* was not detected in any samples [29]. It is rarely reported outbreaks of seafood-related yersiniosis [87]. However, if *Y. enterocolitica* was to contaminate seaweed food products, it is likely that it could grow under refrigeration [88]. 

(ii) *Clostridium* spp. was detected in 8.4% of semi-processed or final seasoned roasted laver collected in processing plants in Korea, but not *C. botulinum* or *C. perfringens* [89]. *C. perfringens* could not be detected in any out of 258 kimbab samples purchased in Korea [29]. 

(iii) *Shigella* spp. (*S. flexneri* and *S. sonnei*) was found in 13.3% of kimbab samples purchased from supermarkets and convenient stores in Korea using a very sensitive method employing enrichment culture prior to PCR [85]. 

(iv) Yeasts and molds were not detected in fresh wild *Palmaria palmata* collected in Northern Ireland [18], nor in *P. palmata* or *Ulva rigida* collected in France [84]. In air-dried samples of *P. palmata* harvested in France, some molds (log 2.7 cfu/g) were found after 126 days of storage in the dark at 12 °C in sealed (not vacuumed) polyethylene bags [90]. The populations of molds/yeast in commercial dried seaweed stored at a relative humidity (RH) of 90% and at 25 °C for 15 days were log 6.42 cfu/g, but significantly lower when stored at RH 70% (log 2.12 cfu/g) and 50% (log 1.35 cfu/g) [91]. Few international standards specify limits for molds and yeast in seaweed products, except for China. According to the General Administration of Quality Supervision, Inspection, and Quarantine in China (AQSIQ), molds must be <300 cfu/g in dried laver products, to ensure food safety [89,92,93]. 

### 2.5. Viruses

Viruses are intracellular obligate parasites, which means they cannot replicate in the environment outside a cell. Although viruses do not multiply in water or in food matrixes, many viruses still pose a risk as food-borne pathogens [94] due to their low infectious dose and robust survival in the environment [95]. Any virus that is shed in feces can potentially transmit via food, but among registered foodborne viruses that cause disease, norovirus (NV) and hepatitis A virus (HAV) are dominating. Norovirus and HAV are responsible for an estimated 20% and 2% of global foodborne illnesses, respectively [94,96,97]. Both NV (*Caliciviridae*) and HAV (*Picornaviridae*) are small, non-enveloped viruses that contain a single stranded RNA as genomes. Noroviruses constitute several genogroups and genotypes and have a broad animal host range but are not considered zoonotic agents. The human NVs are found in genogroup I and II. Hepatitis A virus is only found in the human intestine and the source of foodborne NV and HAV is, therefore, human feces that contaminates through irrigation water, sewage, surfaces, and handling of food. As non-filter feeders, seaweed may not be considered high risk for food-borne viral transmission compared to e.g., oysters. Histo blood group antigens (HBGA) are cellular intestinal carbohydrate receptors for NV and are also found in oysters [98] and on some leafy greens [99], These products are often connected with outbreaks of NV disease, probably due to the binding of NV to the HBGA. Whether these receptors could also be present on seagrass is not known. However, the disease caused by NV has been linked to seaweed. In 2017, more than 2000 persons in Japan got ill with NV gastroenteritis from eating dried shredded seaweed (nori) [28,100]. The nori was used as a topping on cooked rice. Investigators suspected contamination of seaweed during the shredding process. The processing company stated that the seaweed had been heat-treated at 240 °C for seven seconds and subsequently submersed in 90 °C water for 2 h but had been handled with bare hands by an infected operator during the subsequent cutting and processing operations. The epidemiologic studies showed that NV maintained infectivity for more than 2 months under dry and ambient temperature conditions. In South Korea, 91 students at two schools got NV disease after consumption of uncooked, vinegar seasoned green seaweed [101]. Vinegar can eliminate some microbes, but NV is resistant to harsh environmental conditions and can remain stable under low pH [102,103]. Investigation of the two outbreaks did not conclude whether the seaweed was contaminated during farming or subsequent washing processes. Further, seaweed imported from China has caused outbreaks in European countries [104]. In Norway, more than 100 people became ill with NV from imported frozen Wakame seaweed served in restaurants. Norovirus was detected both in patient stool and in the seaweed. Outbreaks in several other European countries were probably linked to this product [104]. Farming of seaweed in sewage-contaminated water and handling of the seaweed are probably the main routes of viral contamination. Thermal processing is an effective strategy in inactivating foodborne viruses and temperatures ≥90 °C for >90 s are generally effective [94]. Properly heated seaweed should, therefore, constitute no risk as a vector for infectious enteric viruses, unless the product is contaminated after this process. On the other hand, viruses remain relatively stable under refrigerated and freezing conditions [94].

### 2.6. Antimicrobial Resistance

Antimicrobial resistance (AMR) is a current worldwide public health concern, where the over- or misuse of antimicrobials in any setting, aquaculture, agriculture, or human medicine, can compromise the successful treatment of bacterial infections [30]. Many antibiotic resistance genes (ARGs) originate from natural environments [105], and environments influenced by anthropogenic activities as waste water discharge and run-off from agricultural land fertilized by animal manure, are considered hotspots for the development and spread of AMR [106]. Bacteria carrying resistance genes can be transmitted between humans, animals, and the environment, including the marine setting [107]. Even though the marine environment has been characterized as a vast reservoir of ARG [108], its role in the development and dissemination of AMR to humans is not well understood. Thus, the literature is scarce on AMR in human pathogens in the marine environment, although previous studies have reported resistance among *E. coli,* members of the genus *Vibrio,* and *Klebsiella* sp. [75,109,110]. 

Lately, increased awareness of food as a carrier of AMR and ARG has been seen [111,112]. The presence of resistant bacteria is documented in the human food supply chain, which represents a potential exposure route and risk to public health [31]. 

Seaweeds can be involved in AMR development and spread by several mechanisms. The first is the selection of AMR bacteria in the environment by antimicrobial products from seaweeds [113]. Secondly, the conditions on the surface of seaweeds provide a stable environment with a high density of bacteria favoring horizontal genetic transfer of ARG [113]. Finally, seaweed can be contaminated by AMR bacteria during harvest, transport, or processing and find their way to the consumer, particularly during consumption in a raw or lightly preserved state.

The relative importance of seaweeds in the possible development and spread of AMR in the environment or as food is by far well described, and further study would be needed.

## 3. Processing and Factors That Control Microbial Growth in Seaweed

Processing methods for preservation are intended to make food edible, palatable, and safe so that it can be used beyond the harvest season. According to the FAO Globefish Research Programme [1], dried seaweed products are today totally dominating the market. However, seaweeds have recently become more widespread in new markets and introduced as an ingredient in a number of new products in the US and European market, and these alternative methods to drying are gaining interest. With the use of seaweeds distributed as raw (fresh or frozen) or minimally processed and intended as an ingredient by the food industry rather than the end, the consumer comes a need for more knowledge on processing. Still, the enhancement of drying technologies due to the increasing focus on sustainable production is of major importance and the food safety aspects must be considered in this perspective. 

### 3.1. Drying

Drying may inhibit all microbial growth including yeast and mold by reducing the water activity (a_W_) to 0.6 or below, while bacteria of relevance are inhibited at much higher a_W_ according to Table 2. The optimal a_W_ for a food product is usually a compromise between several priorities. At a_W_ below 0.30, lipid oxidation will occur and Maillard reaction has an optimum at a_W_ = 0.65 [114] and high-temperature drying should therefore not be used down to this level. Seaweed processors will, in general, avoid drying to lower moisture content than needed for the preservation of the products as the weight loss and drying costs represent a direct economic loss. Determination of the optimal a_W_ and moisture content is therefore essential. To achieve this, the relationship between the moisture content of the seaweeds and a_W_ has to be determined but literature on this has not been found. Some correlations have been documented for other foods, e.g., algae and fish by the method of da Silva et al. [115]. A more fundamental understanding of the relation of water content, a_W,_ and water structure in foods has been presented by Mathlouthi, 2001 [114] who proposed a method for determining the correlations and validated it for sugars. 

The surface-to-volume ratio is very high for most seaweeds and the drying time is relatively short which makes it feasible to dry at low temperatures (<< 60 °C) without risking microbial growth during drying. Typical low-temperature drying methods are sun drying and drying with dehumidified air but may also be achieved by electromagnetic drying by microwaves or radio frequency. The latter may also be used for high-temperature drying alone or in combination with hot air drying, infrared drying, or alternatively by superheated steam drying. These high-temperature drying methods may be designed to inactivate both bacteria and spores of bacteria. This may be of interest when the dried seaweeds are intended for use as ingredients in moist foods intended to have a shelf life after the addition of the seaweeds. 

### 3.2. Thermal Processing

Blanching and boiling of seaweeds are done for several purposes including the inactivation of microorganisms and inactivating inherent enzymes causing the breakdown of the product. Brown seaweeds commonly have an unacceptable high concentration of iodine which may be reduced by up to 94% by boiling for a few minutes. However, boiling causes loss of flavonoids and water-soluble nutrients which limits the prevalence [116]. 

There are currently few thermally processed seaweed products in the market compared to dried seaweed, but they are found as ingredients in canned (e.g., mackerel in tomato sauce), pasteurized (e.g., fish burgers), fried and boiled (e.g., soup) products. 

The edible seaweed laver (*Porphyra umbilicalis*), commonly named nori, is cultivated and consumed in East Asia [117] and is one of the most commonly used seaweeds for human consumption. It is manufactured as dried and/or processed products and is in great demand as side dishes and snacks. Dried laver may be a contamination source to kimbab and in rolled sushi [118], but Choi et al., 2014 [89] showed that heat-processed laver (260 to 400 °C, 2 to 10 s) had reduced aerobic bacterial counts, and no non-spore-forming pathogens (coliforms, *L. monocytogenes*, *S. aureus*, *Salmonella* spp. and *V. parahaemolyticus*). 

Thermoresistant *B. cereus* was occasionally found and suggested as a target organism in the risk assessment. From the heat treatments in the study of Blikra et al., 2019 [21], they also suggested the need to control the growth of toxin-producing spore-forming bacteria such as *B. licheniformis* and *B. pumilus* during handling and storage. The heat inactivation kinetics of *B. cereus* is well described for several growth media but not specifically for seaweeds. The decimal reduction time at 95 °C is typically found to be around 10 min or higher for *B. cereus* in agar [119]. These values are not necessarily of relevance to seaweeds, as only less heat-stable spore forms have been documented so far. Gupta et al., 2010 [40] found that heat treatment of 85 °C for 15 min inactivated all microorganisms except spore formers which germinated after this treatment and resulted in bacterial counts as high as log 7 cfu/g. They further reported that heat treatment of 95 °C for 15 min inactivated all surface microflora. 

Seaweeds have a low thermal conductivity compared to fish and the leaves may clump together in many layers, resulting in a configuration where it is hard to predict the exact heat load and therefore the heat inactivation of microorganisms may be difficult to assess as well. A popular method of boiling and at the same time increasing the shelf life is vacuum packaging in a sealed pouch or container before the heat treatment, but this can also be challenging. Akomea-Frempong et al., 2021 [120] vacuum-packed sugar kelp in bags of 350 g and blanched at 100 °C for 3 min and found no significant impact of the heat treatment with respect to the microflora, possibly because of poor heat penetration. The vacuum packaging of thin leaves is challenging, and residual air may be observed. Residual air in pouches may lead to poor heat transfer and cold spots [121] where microorganisms may survive. Due to the aforementioned information, it is crucial to both perform heat penetration measurements and demonstrate the heat inactivation of a selected target organism by challenge testing. 

### 3.3. Fermentation

Successful fermentation stabilizes the raw seaweed biomass by producing lactic acid and quickly reducing the pH of the seaweeds to below 4.3, where most potentially pathogenic bacteria are inactivated at refrigeration temperatures (pH 3.7 for ambient temperatures, cf. Table 2). Lactic acid fermentation of seaweed is a recent strategy and quite limited information is available on culture conditions [122,123]. The absence of natural lactic acid bacteria (LAB) microflora and simple sugars in most seaweeds, as opposed to terrestrial plants, may have limited development of this technique in the former [123]. Fermentation may be a preferred processing technique for seaweeds because several seaweed species are sensitive to both thermal treatment and freezing that often diminishes the sensorial properties, appearance, and nutritional value of the products. However, as shown by Uchida et al., 2007 [122], LAB fermentation of *Undaria pinnatifida* is not straightforward due to the selective survival of potential pathogenic spore-forming *Bacillus* spp. through the drying process that could not be effectively outcompeted by the LAB starter culture during fermentation. When cultivated seaweed was mixed with sauerkraut at a ratio of up to 1:1, LAB fermentation proved successful by resulting in sufficiently low pH and thus maintained acceptable microbial and sensorial quality up to 60 days post-inoculation [123]. Heat treatment (95 °C for 15 min) followed by fermentation using a commercial *Lactobacillus plantarum* starter culture led to a drop in pH and stabilization at pH 4.5 after 40 h in *Saccharina latissima* [124], and although this is above the limit set at 4.3 in regards to the growth of *B. cereus* (Table 2), no colonies with the morphology of *B. cereus* were observed [124]. 

### 3.4. Freezing

During the freezing of seaweeds, most of the water content is immobilized around the freezing point of seawater which depends on the salt content of the actual seaweed, usually between 0 °C and −2 °C. Water bound to other molecules has shown a freezing depression in the range −12 °C to −25 °C before rinsing, but after proper rinsing and loss of salts, the freezing point is increased to 0 °C [125]. This change in the freezing point is important for the availability of water to microorganisms. 

There is surprisingly little literature available on the freezing of seaweeds, possibly due to the limited changes during long-time frozen storage. Del Olmo, Pico, and Nunez, 2019 [126] documented 72% retention of polyphenols and 79% retention of antioxidant capacity after 180 days of storage at −24 °C. While freezing to a temperature below −25 °C is an effective measure to protect against microbial growth during storage, the damage to the cell structure during freezing and thawing may make the plant more accessible to microorganisms after thawing. During thawing, the drip loss released from the seaweeds may provide a pathway for the microorganisms. 

Rapid freezing and thawing are recommended to minimize the risk of microbial growth as well as to limit the drip loss as much as possible. This may be achieved by thin layer band freezers or in vertical plate freezers if the width of the blocks is limited to keep the freezing time below a few hours. Block freezing on racks without air circulation and other methods needing several days to freeze the product will be less effective than rapid freezing with respect to food safety. 

### 3.5. Salting

Salting may be very effective when harvesting large amounts of seaweeds over a few days compared to boilers, dryers, or other energy-demanding utensils with limited capacity. Wei et al., 2021 [127] found that a salinity above 20% preserved several seaweed species even at room temperature storage, and refrigeration preservation by the salinity of 10% worked as well. However, the high salt content limits the further use in several products and the amount of seaweed that can be used without further processing. Such processing could be drying, but just like for freezing, the producers would like to avoid steps that are not necessary before the drying to avoid extra costs. 

### 3.6. Gamma Radiation 

Gamma radiation (up to 10 kGy) can reduce the titer of norovirus in green (*Capsosiphon fulvescens*) and brown (*Hizika fusiforme*) seaweeds by >2 log plaque-forming units (pfu) per mL without affecting color, and should be considered in seaweed processing and distribution [128]. The radiation sensitivity (D-10-values or the dose required to inactivate 90% of a population) of *Salmonella* Typhimurium, *E. coli*, *S. aureus*, and *Listeria ivanovii*, ranged from 0.27 to 0. 44 kGy in dried seaweed and the growth of all four test organisms inoculated (log 8 cfu/g) was inhibited by irradiation during 24 h of post-irradiation storage regardless of the temperature (10, 20, and 30 °C) after 2 to 3 kGy treatment, indicating gamma radiation as an effective measure to improve food safety in e.g., kimbab [129]. Research on other kinds of seafood shows that gamma radiation effectively decimates e.g., *S. aureus*, *B. cereus*, *Salmonella* Typhimurium, *L. monocytogenes, V. parahemolyticus,* and *E. coli* in fish [130], frozen seafood [131], and fermented oysters [132]. Since gamma radiation does not affect sensory quality, it may offer an alternative processing method for food products that are sensitive to thermal processing, as many seaweeds are, and thus improve the microbiological quality of these products and reduce the risk of food-borne pathogens. 

### 3.7. Emerging Trends (Other Novel Technologies)

Several technologies have been suggested for the processing of macroalgae but have not yet, to our knowledge, been used in commercial production. 

Plasma activated Water (PAW) is a technology currently not recognized as safe for the treatment of foods but is still being investigated in laboratories both for disinfection of utensils and packaging and even directly on foods. PAW has proven its ability to inactivate several microorganisms and even spores of *B. cereus* have been totally inactivated [133]. By rinsing seaweeds after harvesting PAW can reduce the surface flora and could hold potential for removing fouling by bryozoa and other organisms feeding on the seaweed. 

High-pressure processing (HPP) is a more established technology and inactivation kinetics of a broad range of microorganisms has been reported for a wide range of foods [134]. HPP has been used for the preservation of kombu and a variety of brown, green, and red seaweeds [126,135] and it has been documented to reduce a wide range of microflora while spores of *Bacillus* sp. remain intact. Further research on the preservation of quality as well as the costs associated with HPP may reveal the commercial potential of the method as the results reported so far are very promising.

Pulsed electric fields (PEF) and ultrasound (US) have been proposed as pre-treatments of macroalgae for enhancing dewatering and extraction of high-value components, e.g., fucoidan, and this research may result in commercially feasible methods for reducing undesired levels of iodine and heavy metals. However, it is also known that these methods to some extent may reduce the microbial surface flora on several foods when used for rinsing.

The inactivation of bacteria by PEF has been proven for several liquid foods. For use on solid foods an electrolyte surrounding the dry matter is necessary. PEF is effective for the inactivation of vegetative cells, especially Gram-negative, but spores have a high resistance [136]. No information on microbial inactivation on seaweed by PEF has been found and neither are reviews of the dielectric properties of seaweeds available. Due to this it is not yet possible to rule out PEF as a potential preservation method for seaweed but is more likely that any inactivation effect would be a side effect of PEF used as pre-processing. 

Park, Song, and Ha, 2014 [137] reported the effect of inactivation of microflora on laver by the US in combination with 200 µg/g NaOCl to reduce contamination of *E. coli* with up to 0.5 log cfu/g and spores of *B. cereus* with up to 1.1 log cfu/g. The US alone is less efficient than the aforementioned combination and is therefore without interest in food safety and durability. However, for many other foods, the combination of water bath pasteurization and the US has shown to be most effective [138]. An example of this has been presented by Silva, 2015 [139] who reduced the decimal reduction time at 100 °C by 50% for *C. perfringens* in meat slurry by pre-treating the spores by the US prior to thermal processing. The same methodology may be used for seaweeds as well, and the increased cook loss would then have to be considered. This cook loss, may on one hand contain heavy metals, which are desired to be removed from the seaweed, but on the other hand, there is a significant loss of nutrients by use of US [140]. 

## 4. Guidelines and Legislation 

The Centre d’Etude et de Valorization des Algues (CEVA) recommended guidelines regarding quantitative limits in dry edible seaweed products, and quantitative limits for seaweed are also introduced in e.g., Korea and China (Table 3). The general principles and requirements of seaweed food safety in the EU are subject to the EU enforced Regulation (EC) no 852/2004 on food hygiene. In many countries, the food manufacturing process is subject to Hazard Analysis and Critical Control Point (HACCP) assessment; a system adopted by the World Health Organization and the Codex Alimentarius Commission as recommended international code of practice for general principles of food hygiene. However, considering the new market trends and novel processing technologies and seaweed food products, guidelines and legislation on specific seaweed food products are still lacking. It is also doubtful whether legislation from one part of the world can be transferred to other areas as well without taking, e.g., biological (seaweed and microbial flora) and environmental (climatic) factors into account. 

## 5. Data Gaps

Increased interest in sustainable seaweed diets has opened for new markets and applications, which necessitates a turn in the research focus from the traditionally dominating drying processes towards novel methods to process and utilize the seaweed raw materials taking bioeconomic principles into account. For example, systematic and published trials with the preservation of seaweeds by fermentation are relatively scarce, and further study on optimal conditions for the process and the effect on pathogenic bacteria and shelf life should be conducted.

Data from Asia on seaweed food safety is abundant, and Europe and the Americas are catching up on research interest concurrent with the market trends and increased consumer demand for seaweed food products. Data from Africa are however scarce, indicative perhaps of the historical and current low levels of commercial interest or value [1].

Seaweeds are densely populated by bacteria on their surfaces, and horizontal gene transfer could occur enhancing the distribution of ARGs. The possible role of seaweeds in the development and spread of AMR in the environment or as food is, by far, well described, and further study would be needed.

Predictive microbiology deals with the study of models for microbial growth and survival under particular environmental conditions and it has been developed and implemented to predict the occurrence and growth of food-borne pathogens. Relatively few predictions are so far carried out for pathogenic bacteria in seaweeds and may reflect lacking data on the support of growth conditions in seaweeds. An exception is modeling on *Staphylococcus* sp.

## 6. Conclusions

The present review has identified pathogenic *Bacillus* spp., *Vibrio* spp., and *Aeromonas* spp. as the main inherent bacteria that are of special concern for the food safety of seaweeds. *Bacillus* spp. forms heat-resistant spores and can produce heat-stable toxins, whereas *Vibrio* and *Aeromonas* spp. can grow under chilled temperatures. Several bacterial species, including *E. coli*, *Salmonella* spp., *S. aureus,* and *L. monocytogenes*, and Norovirus and Hepatitis A virus, are considered as potential food safety concerns, predominantly by virtue of recontamination during processing. Some other pathogenic bacteria, e.g., *Campylobacter* spp., *Clostridium* spp., *Shigella* spp., and yeast and molds, are considered as seaweed associated and can on rare occasions lead to food poisoning, however presumably because of gross violations of food safety protocol. Further studies and risk analysis, and updated guidelines concerning food safety of both wild-harvested and cultivated seaweed, are necessary. Several preservation technologies are available, but traditional technologies like drying, freezing, and heat treatments, like blanching and pasteurization are still the most obvious ways to achieve food safety. However, due to the energy demands, these processes will continue to be challenged by novel methods. In Asia, where seaweeds have historically been a more important part of the everyday cuisine than in many western countries, expertise on seaweed food preparation and processing has accumulated for generations, and the legislative framework for food safety may have been better incorporated to also include seaweed. Exchange of experiences between East and West will certainly lead to increased knowledge and improved food safety for the benefit of society and consumers. However, biological (seaweed and microbial flora), environmental (climatic), and cultural differences must be accounted for.

## Figures and Tables

**Table 1:** Reported viable bacteria counts for selected seaweed species of relevance for human consumption.

Seaweed Species [Wild (w), Cultivated (c), or Unknown (u)]	Sampling Location/Region	Month and Year of Sampling	Bacterial Density (Log cfu/g) *	Method [PC; Platecounting, M; Microscopy]	Reference
*Polysiphonia [Vertebrata] lanosa* (Sea Truffle) (w)	Point Lepreau, Bay of Fundy, New Brunswick, Canada	May–September, 1964	~6–7	PC: Seawater Tryptone Soy Agar, Aerobic, 10 °C	[12]
*Ascophyllum nodosum* (w)			~4–7		
*Ascophyllum nodosum* (w)	Nahant, Massachusetts, USA	February, 1976	~8 ^†^	M: Scanning Electron Microscopy	[14]
*Palmaria palmata* (c)	Bristol, ME, USA	February (year not given)	~3	PC: Petrifilm Aerobic Count Plate, 37 °C	[15]
*Gracilaria tikvahiae* (c)		September (year not given)	~4		
*Gracillaria* spp. (u)	Mactan Island, Cebu, Philippines	March, 1996	~8–9	M: DAPI-staining	[16]
*Kappaphycus alvarezii* (c)	Uranouchi Inlet, Tosa Bay, Southern Japan	Not given	~5		
*Ulva lactuca* (c)	Izmir, Turkey	March and April, 2005	4.94	PC: Plate Count Agar, aerobic, 37 °C	[17]
*Palmaria palmata* (w)	Cushendall, County Antrim, Northern Ireland (55 °N)	Not given	5.11	PC: Plate Count Agar, aerobic, 30 °C	[18]
*Caulerpa lentillifera* (Sea grape) (c)	Okinawa, Japan	August–September, 2006	~7	PC: Marine Agar, aerobic, 25 °C	[19]
*Gracilaria changii* (c)	Mengabang Telipot, Malaysia	December, 2007	8.46	PC: Tryptic soy agar with 2% NaCl, room temperature	[20]
*Alaria esculenta* (c)	West Norway (61° N)	March and April, 2016	2.01	PC: Marine Agar, aerobic, 25 °C	[21]
*Saccharina latissima* (c)			1.10		
*Alaria esculenta* (c)	Port-a-Bhuiltin seaweed farm, Scotland	2019	5.2	PC: Marine Agar, aerobic, 30 °C	[8]
		2020	3.2		
*Saccharina latissima* (c)		2019	3.7		
		2020	<2		
*Laminaria hyperborea* (w)	Outside Bergen, West Norway (60° N)	March, 2007	~4 ^†^	M: DAPI-staining	[10]
		May, 2007	~6 ^†^		
		July–February, 2007	~7 ^†^		
*Fucus serratus* (w)	Millport, Scotland	July and August, 1990	7.7 ^†^	M: Scanning Electron Microscopy	[22]
*Porphyra umbilicalis* (w)			7.2 ^†^		
*Ulva lactuca* (w)			7.6 ^†^		
*Chaetomorpha* sp. (w)	Vellar Estuary, Parangipettai, India	September, 1978	6.54	PC: Estuarine Peptone Yeast Extract Agar	[11]
		October, 1978	6.96		
		March, 1979	6.58		
		April, 1979	6.24		
		May, 1979	6.06		
*Enteromorpha* sp. (w)		September, 1978	6.50		
		October, 1978	7.27		
		March, 1979	6.47		
		April, 1979	6.13		
		May, 1979	6.05		
*Hypnea* sp. (w)		September, 1978	6.63		
		October, 1978	7.14		
		March, 1979	6.61		
		April, 1979	6.17		
		May, 1979	6.06		
*Chondrus crispus* (u)	A Coruna Province, North-western Spain	Not given	4.21	PC: Marine Agar, aerobic, 25 °C	[23]
*Himanthalia elongata* (u)			1.67		
*Laminaria ocroleuca* (u)			4.36		
*Palmaria palmata* (u)			3.71		
*Porphyra umbillicalis* (u)			3.56		
*Saccharina latissima* (u)			3.09		
*Ulva lactuca* (u)			3.15		
*Undaria pinnatifida* (u)			4.99		
*Undaria pinnatifida* (u)	Commercial products purchased in Italy	Not given	3.39–5.49	PC: Marine Agar, aerobic, 30 °C	[24]
*Palmaria palmata* (u)			3.09–5.31		
*Laminaria* spp. (u)			2.23–4.54		
*Ulva* spp. (u)			2.88–5.58		
*Hizikia fusiformis* (u)			2.23–4.35		
*Alaria esculenta* (c)	West Norway (61° N)	March, 2015	3.59	PC: Marine Agar, aerobic, 25 °C	Unpublished results, the authors
*Laminaria digitata* (c)			2.79		
*Saccharina latissima* (c)			2.75		
*Saccharina latissima* (w)	West Norway	February, 2020	3.63	PC: Plate Count Agar, aerobic, 30 °C	

* cfu; colony forming units. ^†^ Given as bacteria/cm^2^.

**Table 2:** Limits for growth under otherwise optimal conditions for some pathogenic bacteria of relevance for seaweeds. The data are relevant for safety and shelf life for traditional and novel seaweed food products under different processing and storage conditions.

Pathogen		Temperature		pH		a_w_ (min)	Max. Water Phase NaCl (%)	Reference
		Min.	Max.	Min.	Max.			
*B. cereus*		4	55	4.3	9.3	0.92	10	[47]
*B. licheniformis*		11–15	50–55	4.6	9.8	0.91	7	[32,48]
*B. pumilus*		>5–15	40–50	≤6 (Some strains grow at 4.5)	≥9.5	<0.96	>10	[35,49]
*B. subtilis*		5.5	55.7	4.8	9.2	0.93	>5–10	[32,50]
*C. botulinum* (growth only, proteolytic)		10	48	4.6	9	0.93	10	[47]
*C. botulinum* (growth only, non-proteolytic)		3.3	45	5.0	9	0.97	5	
*C. perfringens*		10	52	5	9	0.93	7	
Pathogenic *E. coli*		6.5	49.4	4	9	0.95	6.5	[47,51,52,53]
*L. monocytogenes*		−0.4	45	4.4	9.4	0.92	10	[47]
*S. aureus*	aerobe	7	50	4	10	0.83	20	
	anaerobe			5.0		0.90		[51]
*Salmonella*		5.2	42.6	3.7	9.5	0.94	8	[47]
*V. cholerae*		10	~44	5.0	~10	0.97	3	[47,54,55,56]
*V. parahaemolyticus*		5	~44	4.8	~11	0.94	8	[47,54,55,56,57]
*V. vulnificus*		10	~44	4.4	~9	0.96	6	[47,57,58]
*Aeromonas hydrophila*		0	42	6	7.2 (optimum)	0.97	5	[47,59]

**Table 3:** Selected standards for microbial load in seaweed food products.

Pathogen	Limit (cfu per g)	Comment	Reference
Aerobe mesophiles	≤10^5^	French guidelines that apply to dry seaweed products	[141]
Coliforms (fecal)	≤10		
Anaerobe sulfite reducers	≤10^2^		
*S. aureus*	≤10^2^		
*C. perfringens*	≤1		
*Salmonella*	Not present per 25 g		
*S. aureus*	<10^2^	Korean legislation that applies to RTE foods, including RTE seaweed	[85,142]
*B. cereus*	<10^3^		
*Salmonella* spp.	0		
*Shigella* spp.	0		
*L. monocytogenes*	0		
Aerobic plate counts	<3 × 10^4^ cfu/g	Chinese hygienic standard for marine algae and algae products. Applies also to dried laver	[89,92,93]
Coliforms	<30 MPN/100 g		
Mold	<300 cfu/g		
*Salmonella* spp.	0		
*Shigella* spp.	0		
*V. parahemolyticus*	0		
*S. aureus*	0		
*E. coli*	<100 cfu/100 g	Guidelines for seaweed collected in Danish waters	[26]
*Salmonella*	Not present per 25 g		
